# Supplementary material for: Immigrant community integration in world cities
Source: PLoS One. 2018 Mar 14;13(3):e0191612. doi: 10.1371/journal.pone.0191612 (PMC5851540; doi:10.1371/journal.pone.0191612)
Supplement: S1 File — This file includes 10 tables (Table A: Number of users and tweets in each city; Table B: Location of the city centers; C: Number of users residing in each city; D: Language aggregation process; E: Local languages in each city; F: Number of residents per language and city; G: Power of Integration of the cities; H: City-Country correspondence; I: Languages and country correspondence; J: Data validation) and 7 figures (A: Number of reliable users as a function of filtering parameters; B: Comparison of clustering methods for the cities; C: Degree and weight distribution of the bipartite networks with and without English; D: Data validation 1; E: Data validation 2; F: Relative error of the entropy as a function of the scale Δx; G: Relative error of the link weights as a function of the scale Δx). (PDF) [file pone.0191612.s001.pdf]

Supporting Information for *Immigrant community integration in world cities*

Fabio Lamanna,[1] Maxime Lenormand,[2] María Henar Salas-Olmedo,[3]
Gustavo Romanillos,[3] Bruno Gonçalves,[4] and José J. Ramasco[1,*]

[1] *Instituto de Física Interdisciplinar y Sistemas Complejos IFISC (CSIC-UIB), Campus UIB, 07122 Palma de Mallorca, Spain*
[2] *Irstea, UMR TETIS, 500 rue JF Breton, 34093 Montpellier, France*
[3] *Departamento de Geografía Humana, Facultad de Geografía e Historia, Universidad Complutense de Madrid, 28040, Madrid, Spain*
[4] *Center for Data Science, New York University, New York, 10011 NY, USA*

Supporting Figures

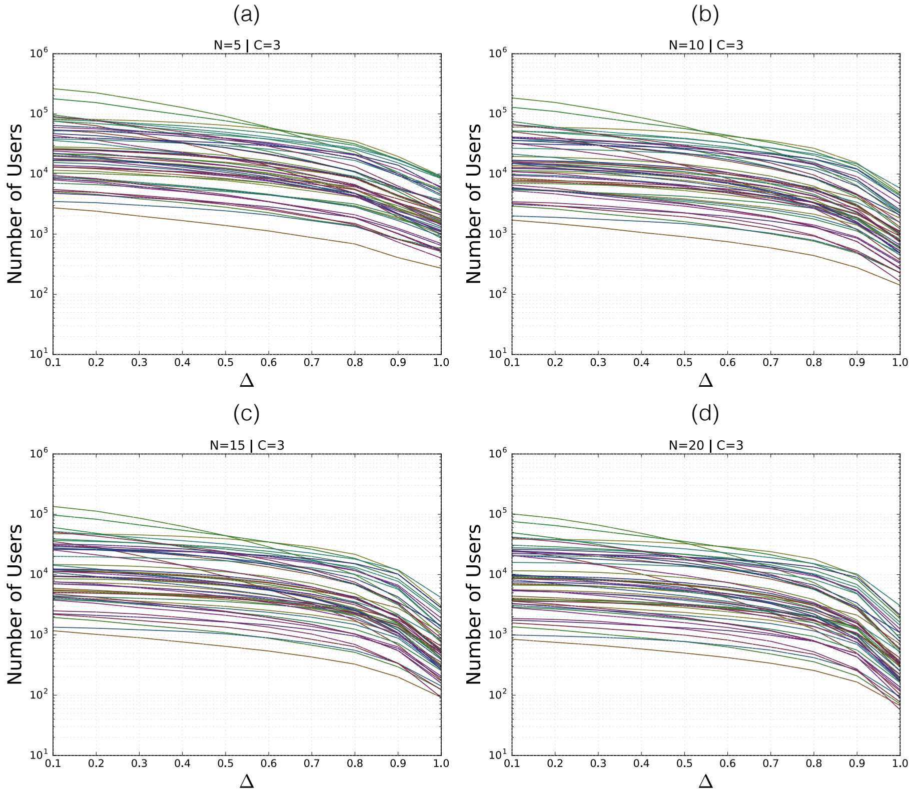

Fig A. Number of reliable users as a function of N and Δ. Each line represents the trend of each city in the number of users according to the ratio between N and the total number of hours of activity for each user (Δ). Set as C=3 the number of months for consecutive activities, (a) refers to N=5, (b) to N=10, (c) to N=15 and (d) to N=20.

*Corresponding author: jramasco@ifisc.uib-csic.es

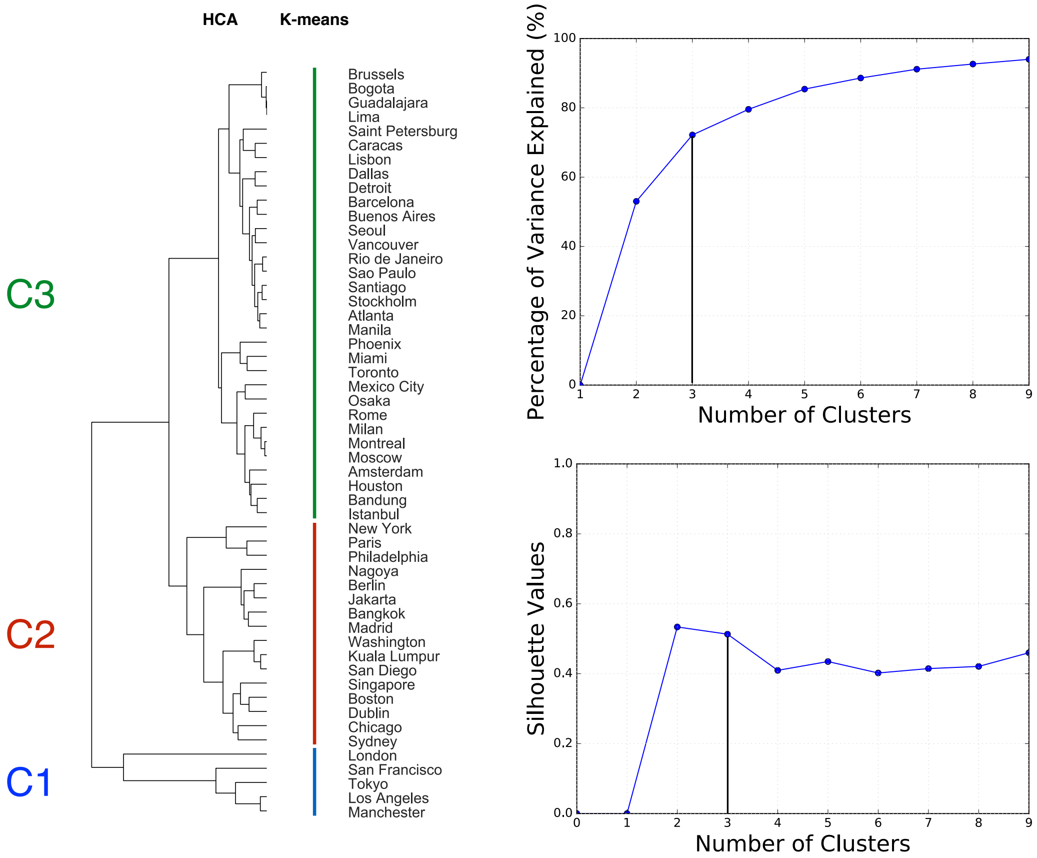

Fig B. Comparison of a k-means and hierarchical clustering algorithms over the vectors of the Bipartite Spatial Integration Network. C1, C2 and C3 are the clusters obtained through both algorithms, choosing as 3 the initial number of clusters to assign to the k-means analysis.

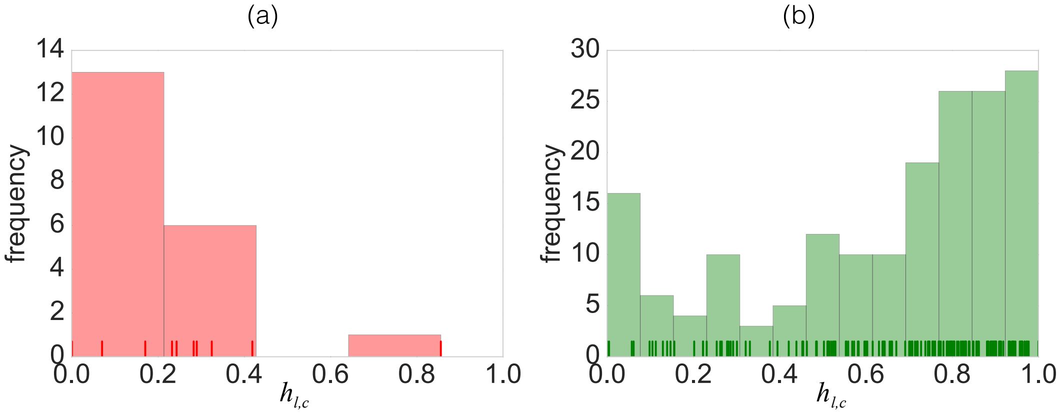

Fig C. Distribution of degree and weights in H with and without English. Distribution of weights for the full network (a) and in the network obtained removing English from the nodes of the Bipartite Spatial Integration Network (b). As shown, English is dominant in the worst links in terms of spatial integration.

Supporting Tables

City	tweets	users
Amsterdam	2082040	175107
Atlanta	4896616	250803
Bandung	7980207	487209
Bangkok	9389888	265672
Barcelona	2249807	168189
Berlin	703551	64360
Bogota	3370596	195830
Boston	3500542	206385
Brussels	1434065	84140
Buenos Aires	18421250	459534
Caracas	1376503	108769
Chicago	5008746	304844
Dallas	6650105	253760
Detroit	3885662	144775
Jakarta	28304891	1260903
Dublin	1516170	89259
Guadalajara	704530	62403
Houston	5337061	193876
Istanbul	19438021	838347
Kuala Lumpur	16730001	412560
Lima	1356562	94234
Lisbon	3341088	57201
London	11167058	698424
Los Angeles	12458292	600476
Madrid	5605452	275857
Manchester	6940211	337083
Manila	19453573	449308

City	tweets	users
Mexico City	4458228	322462
Miami	3716735	241676
Milan	1493614	103383
Montreal	631844	52851
Moscow	2805144	141589
Nagoya	3941030	162606
New York	12960258	734100
Osaka	10607046	351628
Paris	10929091	335553
Philadelphia	5841765	247875
Phoenix	2970897	136501
Rio de Janeiro	20907590	295565
Rome	1240080	96213
Saint Petersburg	1124887	63828
San Diego	2267882	164061
San Francisco	4615005	284215
Santiago	3022229	148786
Sao Paulo	18744302	371032
Seoul	1338750	118509
Singapore	7530611	292571
Stockholm	784807	51729
Sydney	1226072	80349
Tokyo	15029229	644683
Toronto	2281968	150786
Vancouver	660837	58984
Washington	6515118	330808

Table A. Number of tweets and users detected in each city after filtering out bots and multi-user accounts.

City	Latitude	Longitude
Amsterdam	52.370216	4.895168
Atlanta	33.748995	-84.387982
Bandung	-6.914744	107.609811
Bangkok	13.727896	100.524123
Barcelona	41.385064	2.173403
Berlin	52.519171	13.406091
Bogota	4.598056	-74.075833
Boston	42.358431	-71.059773
Brussels	50.85034	4.35171
Buenos Aires	-34.603723	-58.381593
Caracas	10.491016	-66.902061
Chicago	41.878114	-87.629798
Dallas	32.78014	-96.800451
Detroit	42.331427	-83.045754
Jakarta	-6.211544	106.845172
Dublin	53.349805	-6.26031
Guadalajara	20.67359	-103.343803
Houston	29.760193	-95.36939
Istanbul	41.00527	28.97696
Kuala Lumpur	3.139003	101.686855
Lima	-12.047816	-77.062203
Lisbon	38.725299	-9.150036
London	51.511214	-0.119824
Los Angeles	33.95	-118.14
Madrid	40.416775	-3.70379
Manchester	53.479324	-2.248485
Manila	14.599512	120.984219

City	Latitude	Longitude
Mexico City	19.42705	-99.127571
Miami	25.788969	-80.226439
Milan	45.465454	9.186516
Montreal	45.50867	-73.553992
Moscow	55.755826	37.6173
Nagoya	35.181446	136.906398
New York	40.714353	-74.005973
Osaka	34.693738	135.502165
Paris	48.856614	2.352222
Philadelphia	39.952335	-75.163789
Phoenix	33.448377	-112.074037
Rio de Janeiro	-22.903539	-43.209587
Rome	41.892916	12.48252
Saint Petersburg	59.93428	30.335099
San Diego	32.715329	-117.157255
San Francisco	37.774929	-122.419416
Santiago	-33.46912	-70.641997
Sao Paulo	-23.548943	-46.638818
Seoul	37.566535	126.977969
Singapore	1.352083	103.819836
Stockholm	59.32893	18.06491
Sydney	-33.867487	151.20699
Tokyo	35.689487	139.691706
Toronto	43.653226	-79.383184
Vancouver	49.261226	-123.113927
Washington	38.907231	-77.036464

Table B. Coordinates of the centers of the frame for each city.

City	Resident Users
Amsterdam	4986
Atlanta	8474
Bandung	27818
Bangkok	25659
Barcelona	9957
Berlin	1301
Bogota	19353
Boston	8989
Brussels	2325
Buenos Aires	48934
Caracas	4613
Chicago	15397
Dallas	15549
Detroit	10652
Jakarta	98997
Dublin	5480
Guadalajara	3459
Houston	11413
Istanbul	101556
Kuala Lumpur	41084
Lima	2003
Lisbon	4321
London	37402
Los Angeles	70592
Madrid	15447
Manchester	23836
Manila	20093

City	Resident Users
Mexico City	18079
Miami	7754
Milan	4243
Montreal	2613
Moscow	9673
Nagoya	7589
NewYork	34325
Osaka	16348
Paris	19757
Philadelphia	13679
Phoenix	9259
Rio de Janeiro	37177
Rome	1994
Saint Petersburg	3819
San Diego	5014
San Francisco	25504
Santiago	10066
Sao Paulo	21862
Seoul	3099
Singapore	20997
Stockholm	2668
Sydney	4751
Tokyo	75929
Toronto	8737
Vancouver	2298
Washington	10147

Table C. Total number of residents users detected in the cities.

Detected language	Aggregated group
Albanian	Albanian
Arabic	Arabic
Belarusian	**East Slavic**
Bosnian	**South Slavic**
Bulgarian	**South Slavic**
Catalan	Catalan
Chinese	Chinese
Croatian	**South Slavic**
Czech	**West Slavic**
Danish	**Northern European**
Dutch	Dutch (including Flemish)
English	English
Faroese	**Northern European**
Finnish	Finnish
French	French
German	German
Greek	Greek
Haitian	Haitian
Hungarian	Hungarian
Icelandic	**Northern European**
Indonesian	Indonesian
Irish	Irish
Italian	Italian
Japanese	Japanese
Javanese	Javanese
Korean	Korean

Detected Language	Aggregated Group
Kurdish	Kurdish
Lettonian	**Baltic**
Lituanian	**Baltic**
Macedonian	**South Slavic**
Malay	Malay
Norwegian	**Northern European**
Polish	**West Slavic**
Portuguese	Portuguese
Romanian	Romanian
Russian	**East Slavic**
Serbian	**South Slavic**
Serbo-Croatian	**South Slavic**
Slovak	**West Slavic**
Slovenian	**South Slavic**
Southern Sotho	Southern Sotho
Spanish	Spanish
Swahili	Swahili
Swedish	**Northern European**
Sundanese	Sundanese
Tagalog	Tagalog
Thai	Thai
Turkish	Turkish
Ukrainian	**East Slavic**
Vietnamese	Vietnamese

Table D. Language aggregation process. A main Aggregated group has been associated to each language detected in the framework, to overlap ”mutually intelligible” issues in the detection.

City	Local Culture
Amsterdam	Dutch
Atlanta	English
Bandung	Indonesian
Bangkok	Thai
Barcelona	Spanish/Catalan
Berlin	German
Bogota	Spanish
Boston	English
Brussels	French/Flemish
Buenos Aires	Spanish
Caracas	Spanish
Chicago	English
Dallas	English
Detroit	English
Jakarta	Indonesian
Dublin	English/Irish
Guadalajara	Spanish
Houston	English
Istanbul	Turkish
Kuala Lumpur	Malay
Lima	Spanish
Lisbon	Portuguese
London	English
Los Angeles	English
Madrid	Spanish
Manchester	English
Manila	Tagalog

City	Local Culture
Mexico City	Spanish
Miami	English
Milan	Italian
Montreal	French/English
Moscow	East-Slavic
Nagoya	Japanese
New York	English
Osaka	Japanese
Paris	French
Philadelphia	English
Phoenix	English
Rio de Janeiro	Portuguese
Rome	Italian
Saint Petersburg	East-Slavic
San Diego	English
San Francisco	English
Santiago	Spanish
Sao Paulo	Portuguese
Seoul	Korean
Singapore	Malay/Chinese/English/Tamil
Stockholm	Northern-European
Sydney	English
Tokyo	Japanese
Toronto	English
Vancouver	English
Washington	English

Table E. Cities and local languages. Each city has been associated to its main local language; Barcelona, Brussels, Dublin, Montreal and Singapore have been related to more than one language due to the coexistence of multiple languages in the same urban area.

	Albanian	Arabic	Catalan	Chinese	Dutch	East-Slavic	English	Finnish	French	German	Greek	Haitian	Hungarian	Indonesian	Irish	Italian	Japanese	Javanese	Korean	Kurdish	Malay	Northern-European	Portuguese	Romanian	South-Slavic	Southern-Sotho	Spanish	Sundanese	Swahili	Tagalog	Thai	Turkish	Uzbek	Vietnamese	West-Slavic
Amsterdam					4383		247		34	51																	55					138			
Atlanta					38		7747		148														144				305								
Bandung							182							21847				116			3132							2484							
Bangkok				55			493		40					47			154		368											47	24339				
Barcelona			1516				193		72														57				7955								
Berlin		43				42	149		30	779																	69					71			
Bogota							45																				19304								
Boston		109			30		7570		156					112		173	31						218				439			39					
Brussels					1449		81		632																							99			
Buenos Aires							46									601							3001				45267								
Caracas																60							278				4231								
Chicago		55					13277		250	197						206						49	160				1006			84		36			31
Dallas		32					13694		222														218				1186		74	69					
Detroit		36					9964									107							108				347			47					
Dublin		57					4232		142	58					327	57					34		190				180			35					31
Guadalajara																											3427								
Houston		44					9808																183				1269			57					
Istanbul		207				33	99		152											432							119					100443			
Jakarta							1932							95798			33	236	52		752							113							
Kuala Lumpur		55		1298			1134							10751					46		27570							62		71					
Lima																											1956								
Lisbon							65																4052				139								
London	92	584			138	99	30742	122	918	357	46		62	414		552	84				216	206	545	118	48		956		131	176	32	419			213
Los Angeles		556		44		100	59637		1159								269		96			323	880				6451			690	39	129		78	86
Madrid							152		148							144							1065	35			13792			36					
Manchester		136			86		21105		322	235				243		291					94	476	284			44	292			74		43			41
Manila							1264		50							37											62			18620					
Mexico City							90		95							144											17674								
Miami							4968		119	81		57				74						249	155				1961			37					
Milan							92		56							3766											147			75					
Montreal		34					896		1410							52							31				122								
Moscow						5816	138																		3511							39	33		
Nagoya				48			44										7303		37				75							41					
New York		101			119	62	27072		723	453				424		505	79					144	590				3491			225		149			60
Osaka				112			99										15852		120												44				
Paris		148				37	292		18250	39						127	36				31		213		41		383					67			
Philadelphia		74					11910		217	212		72				201					112		217				475			73					33
Phoenix							8264		92	117						66							99				532			47					
Rio de Janeiro									70							216							36050				802								
Rome							124		32							1585											105			54					
Saint Petersburg						2180	46																		1540										
San Diego		142					3869		59	56				46									67				660			44					
San Francisco		75		49	84	45	20980		614					336		287	149		30		89	110	347				1611			357		66		59	161
Santiago							41		35														396				9578								
Sao Paulo									64							154							20970				605								
Seoul							188							47			140		2426												123				
Singapore				1040			3574		75	62				5222			57	48	84		10349		40				46	58		238					
Stockholm							157															2325					46					34			
Sydney		55					3820		100	46				81		87	69				33		66				103			99		39			
Tokyo		40		549		47	938		149	47				157		50	72865		429		59		55				120			196	160				
Toronto		83			35	32	6951		264	94						140	94						178	56			353			238		64			51
Vancouver							1808		150								54						51				78								
Washington		128					8520		200	154											59		168				676			88					

Table F. Number of resident users per language and per city.

Cluster	City	Q1	Q2	Q3	IQR	P_c
C1	London	0.81	0.91	0.95	0.13	0.789
C1	Manchester	0.91	0.95	0.96	0.06	0.543
C1	Los Angeles	0.87	0.93	0.96	0.09	0.518
C1	San Francisco	0.77	0.83	0.92	0.15	0.522
C1	Tokyo	0.71	0.80	0.87	0.16	0.413
C2	Philadelphia	0.88	0.90	0.92	0.04	0.375
C2	Paris	0.76	0.81	0.90	0.14	0.336
C2	Singapore	0.81	0.86	0.95	0.15	0.319
C2	New York	0.31	0.64	0.85	0.54	0.180
C2	Kuala Lumpur	0.83	0.87	0.90	0.07	0.246
C2	San Diego	0.82	0.88	0.93	0.12	0.236
C2	Boston	0.65	0.80	0.88	0.23	0.241
C2	Chicago	0.53	0.82	0.84	0.31	0.247
C2	Dublin	0.57	0.79	0.87	0.29	0.220
C2	Sydney	0.27	0.65	0.75	0.48	0.161
C2	Washington	0.72	0.82	0.84	0.13	0.217
C2	Madrid	0.61	0.91	0.94	0.33	0.159
C2	Nagoya	0.76	0.86	0.97	0.22	0.146
C2	Bangkok	0.40	0.77	0.84	0.43	0.133
C2	Berlin	0.44	0.77	0.90	0.46	0.108
C2	Jakarta	0.42	0.63	0.82	0.40	0.099
C3	Amsterdam	0.30	0.52	0.74	0.44	0.063
C3	Atlanta	0.10	0.21	0.41	0.31	0.026
C3	Bandung	0.36	0.66	0.77	0.41	0.068
C3	Barcelona	0.36	0.60	0.80	0.44	0.044
C3	Bogota	0.25	0.50	0.75	0.50	0.011
C3	Brussels	0.12	0.24	0.62	0.50	0.011
C3	Buenos Aires	0.23	0.51	0.80	0.57	0.029
C3	Caracas	0.25	0.50	0.75	0.50	0.022
C3	Dallas	0.19	0.34	0.40	0.20	0.071
C3	Detroit	0.19	0.39	0.45	0.26	0.064
C3	Guadalajara	1.00	1.00	1.00	0.00	0.000
C3	Houston	0.51	0.57	0.63	0.12	0.087
C3	Istanbul	0.16	0.57	0.69	0.52	0.071
C3	Lima	1.00	1.00	1.00	0.00	0.000
C3	Lisbon	0.16	0.32	0.66	0.50	0.014
C3	Manila	0.11	0.22	0.53	0.41	0.023
C3	Mexico City	0.54	0.74	0.82	0.28	0.070
C3	Miami	0.27	0.41	0.43	0.16	0.121
C3	Milan	0.57	0.76	0.78	0.21	0.103
C3	Montreal	0.61	0.69	0.72	0.11	0.107
C3	Moscow	0.66	0.72	0.76	0.10	0.113
C3	Osaka	0.25	0.83	0.99	0.75	0.037
C3	Phoenix	0.41	0.47	0.56	0.15	0.105
C3	Rio de Janeiro	0.33	0.47	0.63	0.29	0.044
C3	Rome	0.78	0.79	0.88	0.10	0.124
C3	Saint Petersburg	0.40	0.80	0.90	0.50	0.035
C3	Santiago	0.21	0.38	0.61	0.40	0.030
C3	Sao Paulo	0.30	0.48	0.67	0.37	0.040
C3	Seoul	0.28	0.29	0.60	0.32	0.034
C3	Stockholm	0.25	0.37	0.56	0.32	0.033
C3	Toronto	0.10	0.33	0.47	0.37	0.117
C3	Vancouver	0.19	0.38	0.46	0.28	0.047

Table G. Power of Integration of Cities.

City	Local Culture
Amsterdam	Netherlands
Atlanta	USA
Bandung	Indonesia
Bangkok	Thailand
Barcelona	Spain
Berlin	Germany
Bogota	Colombia
Boston	USA
Brussels	Belgium
Buenos Aires	Argentina
Caracas	Venezuela
Chicago	USA
Dallas	USA
Detroit	USA
Jakarta	Indonesia
Dublin	Ireland
Guadalajara	Mexico
Houston	USA
Istanbul	Turkey
Kuala Lumpur	Malaysia
Lima	Per
Lisbon	Portugal
London	UK
Los Angeles	USA
Madrid	Spain
Manchester	UK
Manila	Philippines
Mexico City	Mexico
Miami	USA
Milan	Italy
Montreal	Canada
Moscow	Russia
Nagoya	Japan
New York	USA
Osaka	Japan
Paris	France
Philadelphia	USA
Phoenix	USA
Rio de Janeiro	Brazil
Rome	Italy
Saint Petersburg	Russia
San Diego	USA
San Francisco	USA
Santiago	Chile
Sao Paulo	Brazil
Seoul	Korea
Singapore	Singapore
Stockholm	Sweden
Sydney	Australia
Tokyo	Japan
Toronto	Canada
Vancouver	Canada
Washington	USA

Table H. City/Country Correspondence.

Evaluation of the migrant communities spatial distribution accuracy

Validation data was extracted from the Continuous Register Statistics of the Municipal Register, regarding the cities of Madrid and Barcelona. The smallest spatial units for this dataset are census tracks, of which the latest available geometrical boundaries for both study areas are the corresponding to 2013. It is well known that census tracks cover all the territory (not only populated areas) and that their size depends on the population density of an area, i.e. the more population density, the smallest the size and vice versa, in order to ensure that all census tracks have a similar number of inhabitants. This means that low density census tracks are larger than those corresponding to the city center, thus integrating non populated territory. For this reason, complementary data about the exact location of the residential areas is needed in order to properly geo-reference population data from census track statistics. In this research, information was extracted from the "Downloads of data and cartography by town" service of the SEC, the point of access to electronic services provided by the Directorate General of Land Registry of Spain. This data was transformed in order to obtain the surface devoted to each land use in each urban parcel. Some data treatment was required in order to obtain the number of people residing in each 500 x 500 m^2 grid cell according to the main language spoken in the country of origin. SI Table shows the correspondence between country of origin and the languages detected in the main part of this paper. It is important to notice that not all the countries in the world are present in the original table.

Language	Country of Origin
German	Germany
South Slavic	Bulgaria
French	France
Italian	Italy
West Slavic	Poland
Portuguese	Portugal, Brazil
English	United Kingdom
Romanian	Romania
East Slavic	Russia, Ukraine
Arabic	Morocco, Algeria
Spanish	Spain, Argentina, Bolivia
	Colombia, Cuba, Chile, Ecuador
	Paraguay, Peru, Dominican Republic
	Uruguay, Venezuela
Chinese	China
Urdu	Pakistan

Table I. Correspondence between languages detected in Twitter users and country of origin.

The second step in data treatment was to locate where people in each census track actually live according with the location of residential land. We selected the blocks containing some surface devoted to residential use from the cadastral dataset. With the use of a Geographic Information System (GIS) we were able to intersect these polygons with the census track boundaries, and to assign the population of each census track to its residential land, proportional to the size of each residential polygon within each census track. Finally, the resulting dataset was intersected with the grid used in the previous parts of this research in order to obtain the estimated number of residents of each language in each grid cell.

Anselin Local Morans I is a well-known statistic that provides information on the location and size of four types of clusters: a) high-high clusters of significant high values of a variable that are surrounded by high variables of the same variable; b) high-low clusters of significant high values of a variable surrounded by low values of the same variable; c) low-high clusters of significant low values of a variable surrounded by high values of the same variable; and d) low-low clusters of significant low values of a variable surrounded by low values of the same variable. While the typical tools available in most GIS software solutions allow for univariate analysis, GeoDa is an open source product that also allows the computation of bivariate analysis [1], thus enabling the identification of spatial clusters in which high values of one variable are surrounded by high values of the second (i.e. lagged) variable (high-high clusters) and so on.

Bivariate global Morans I (SJ Table) indicates the existence of positive spatial autocorrelation between the location of tweets and residential areas. In general terms, there is a high positive spatial correlation in both study areas (Morans $I = 0.6$). The z and p values have been evaluated through 99 permutations. This value remains high for local language (Spanish in Madrid and Spanish and Catalan in Barcelona). The spatial autocorrelation of foreign languages is a bit lower, which might be in part due to the inconsistencies between Twitter language and available countries of origin in the official statistics (i.e. United Kingdom is the only country of origin for English speakers and so are Morocco and Algeria for Arabic languages). Anyway, Arabic is the only language whose tweets show a random spatial pattern in relation with the location of resident population from Morocco or Algeria in both cities, whereas tweets in English in Barcelona and tweets in Portuguese in Madrid are highly

Language	City	I	Z-value	pseudo p-value	Spatial Autocorrelation
Total	Barcelona	0,63	236,51	0,01	Positive
	Madrid	0,62	268,62	0,01	Positive
Spanish	Barcelona	0,62	216,99	0,01	Positive
	Madrid	0,62	267,29	0,01	Positive
English	Barcelona	0,50	230,53	0,01	Positive
	Madrid	0,38	190,62	0,01	Positive
French	Barcelona	0,37	151,51	0,01	Positive
	Madrid	0,32	159,25	0,01	Positive
Italian	Barcelona	0,28	125,84	0,01	Positive
	Madrid	0,26	146,32	0,01	Positive
Portuguese	Barcelona	0,32	151,20	0,01	Positive
	Madrid	0,44	204,95	0,01	Positive
Arabic	Barcelona	0,08	89,88	0,01	Random
	Madrid	0,07	41,50	0,01	Random
East-Slavic	Barcelona	0,21	112,83	0,01	Positive
	Madrid	0,06	37,66	0,01	Random

Table J. Data Validation. Global Moran's I.

positively spatially correlated with resident population from the UK and Portugal or Brazil, respectively.

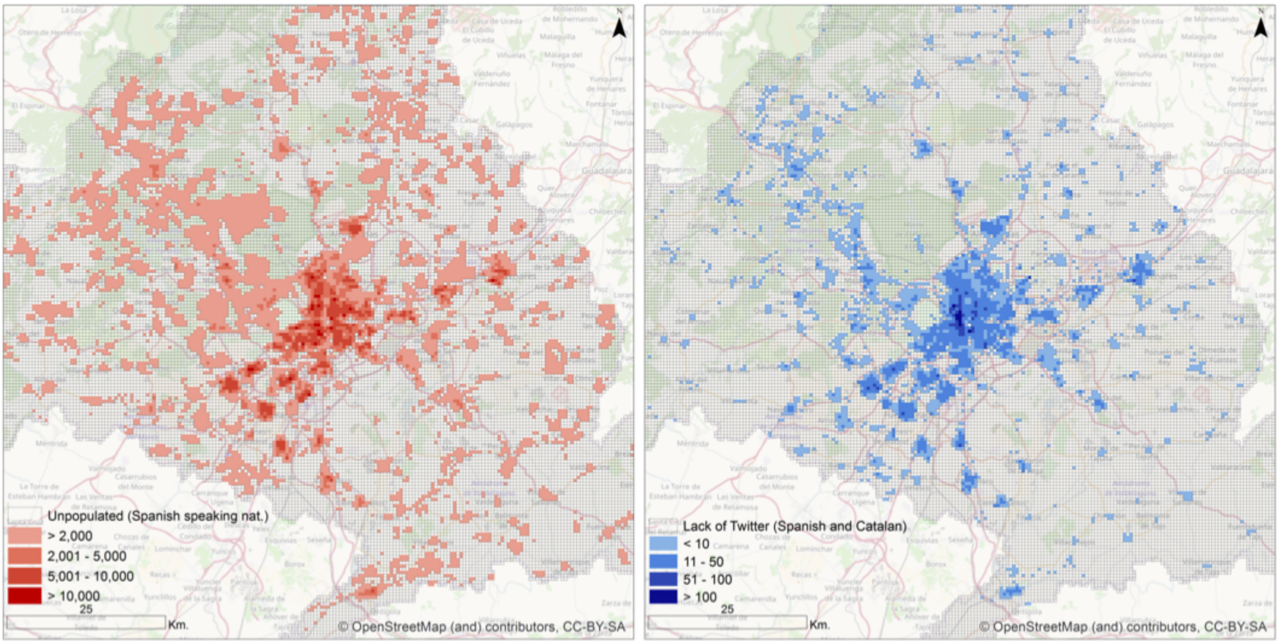

Fig D. Data Validation (1/2). Distribution of Spanish native users in Madrid, according to official statistics and to our framework of language detection process.

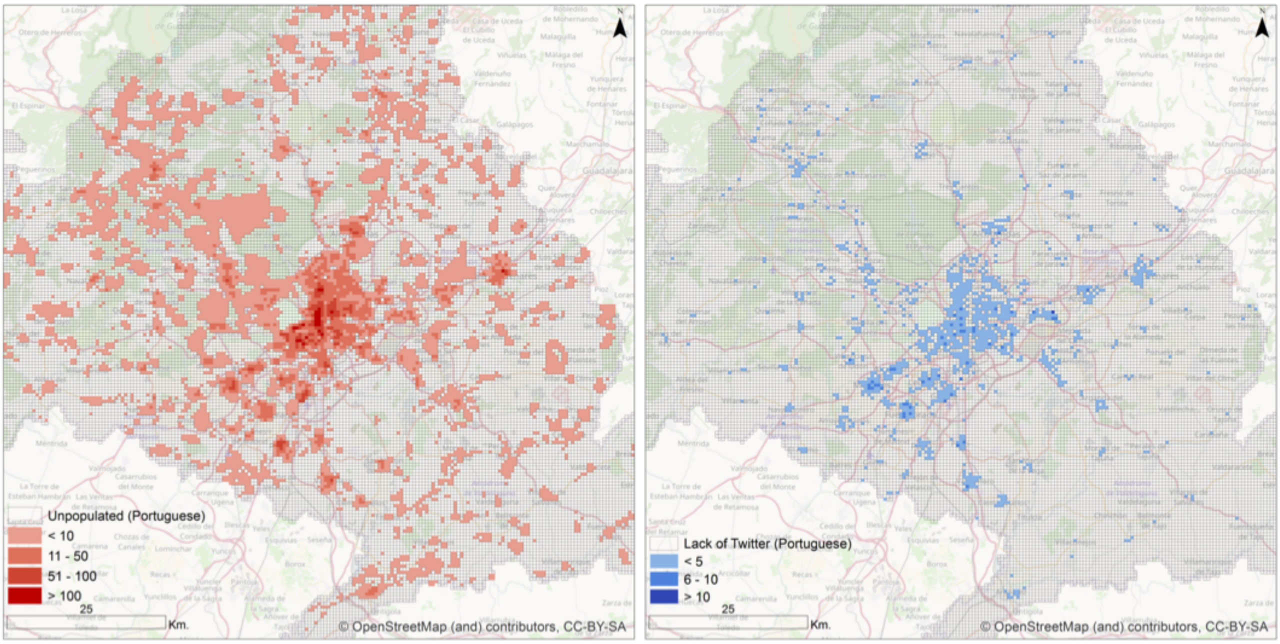

Fig E. Data Validation (2/2). Distribution of Portuguese native users in Madrid, according to official statistics and to our framework of language detection process.

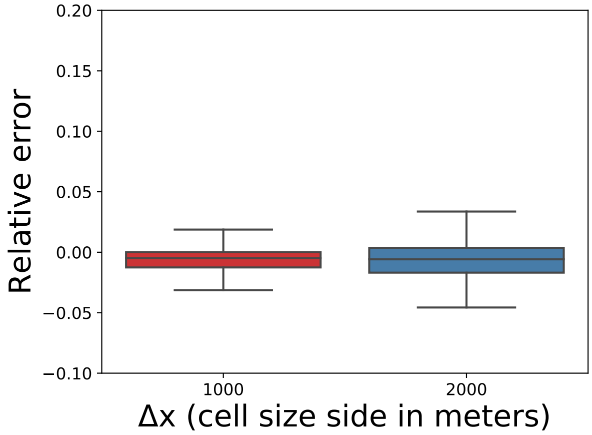

Fig F. Relative error of the spatial entropy in function of Δx. Box plots of the relative change $\epsilon_{l,c}$ of the link weights in the bipartite spatial integration network taking as reference the unit-like Δx as the cell side frame of 500 meters, with respect of 4 and 16 times the Δx for cell side sizes of 1000 and 2000 meters, respectively.

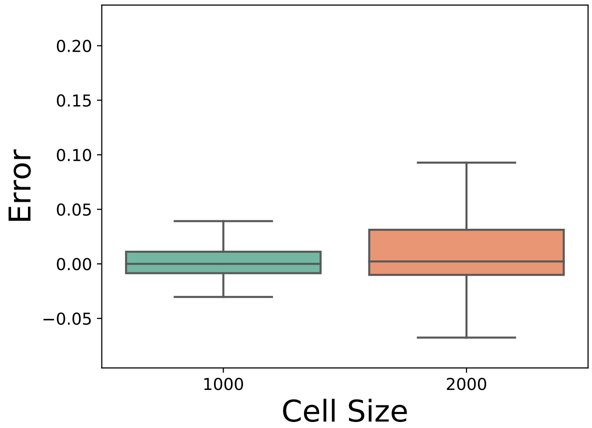

Fig G. Relative error of link weights in function of the cell side size. Box plots of the relative change $\epsilon_{l,c}$ of the link weights in the bipartite spatial integration network taking as reference the 500 meters cell side frame.

References

[1] Anselin L, Syabri I, Kho Y. GeoDa: An introduction to spatial data analysis. Geographical Analysis. 2006;38(1):5–22. doi:10.1111/j.0016-7363.2005.00671.x.
