# Supplementary material for: Immigrant community integration in world cities
Source: PLoS One. 2018 Mar 14;13(3):e0191612. doi: 10.1371/journal.pone.0191612 (PMC5851540; doi:10.1371/journal.pone.0191612)
Supplement: S2 File — (PDF) [file pone.0191612.s002.pdf]

This is an example of the python code used to query the Twitter streaming API in a certain geographical BOX characterized by two limits in latitude y0 and y1, and two in longitude x0 and x1. This code can be used as an illustrative example but it is NOT meant for reproducibility. Twitter developers may change the format of the queries at any moment. The updated information on how to access the Twitter API can be found in the user guide at https://developer.twitter.com/en/docs

```
from tweepy import Stream, OAuthHandler
from tweepy.streaming import StreamListener

CONSUMER_KEY = ''
CONSUMER_SECRET = ''
ACCESS_KEY = ''
ACCESS_SECRET = ''
BOX = [x0, y0, x1, y1]

class MyStreamListener(StreamListener):
    def on_status(self, status):
        print(status)


if __name__ == '__main__':
    auth = OAuthHandler(CONSUMER_KEY, CONSUMER_SECRET)
    auth.set_access_token(ACCESS_KEY, ACCESS_SECRET)

    listen = MyStreamListener()
    stream = Stream(auth, listen, gzip=True)
    stream.filter(locations=BOX)
```
